# The Mixture of Natural Products SH003 Exerts Anti-Melanoma Effects through the Modulation of PD-L1 in B16F10 Cells

**DOI:** 10.3390/nu15122790

**Published:** 2023-06-18

**Authors:** Na-Ra Han, Hi-Joon Park, Seong-Gyu Ko, Phil-Dong Moon

**Affiliations:** 1 College of Korean Medicine, Kyung Hee University, Seoul 02447, Republic of Korea; nrhan@khu.ac.kr; 2 Korean Medicine-Based Drug Repositioning Cancer Research Center, College of Korean Medicine, Kyung Hee University, Seoul 02447, Republic of Korea; epiko@khu.ac.kr; 3 Department of Anatomy & Information Sciences, College of Korean Medicine, Kyung Hee University, Seoul 02447, Republic of Korea; acufind@khu.ac.kr; 4 Department of Preventive Medicine, College of Korean Medicine, Kyung Hee University, Seoul 02447, Republic of Korea; 5 Center for Converging Humanities, Kyung Hee University, Seoul 02447, Republic of Korea

**Keywords:** natural products, SH003, formononetin, melanoma, B16F10 cells, PD-L1, CTLL-2 cells

## Abstract

Melanoma is the most invasive and lethal skin cancer. Recently, PD-1/PD-L1 pathway modulation has been applied to cancer therapy due to its remarkable clinical efficacy. SH003, a mixture of natural products derived from *Astragalus membranaceus*, *Angelica gigas*, and *Trichosanthes kirilowii*, and formononetin (FMN), an active constituent of SH003, exhibit anti-cancer and anti-oxidant properties. However, few studies have reported on the anti-melanoma activities of SH003 and FMN. This work aimed to elucidate the anti-melanoma effects of SH003 and FMN through the PD-1/PD-L1 pathway, using B16F10 cells and CTLL-2 cells. Results showed that SH003 and FMN reduced melanin content and tyrosinase activity induced by α-MSH. Moreover, SH003 and FMN suppressed B16F10 growth and arrested cells at the G2/M phase. SH003 and FMN also led to cell apoptosis with increases in PARP and caspase-3 activation. The pro-apoptotic effects were further enhanced when combined with cisplatin. In addition, SH003 and FMN reversed the increased PD-L1 and STAT1 phosphorylation levels induced by cisplatin in the presence of IFN-γ. SH003 and FMN also enhanced the cytotoxicity of CTLL-2 cells against B16F10 cells. Therefore, the mixture of natural products SH003 demonstrates therapeutic potential in cancer treatment by exerting anti-melanoma effects through the PD-1/PD-L1 pathway.

## 1. Introduction

Melanoma is the deadliest form of skin cancer, and it poses significant clinical and financial challenges [1]. Following the introduction of new therapies such as immune checkpoint inhibitors, deaths from melanoma in the United States dropped sharply by 6.4% per year on average from 2013 to 2017 [2]. One of the most important checkpoint pathways in the tumor microenvironment is the inhibition of tumor-induced immunity regulated by programmed cell death protein 1 (PD-1) and programmed death ligand 1 (PD-L1) [3]. Activation of the PD-1/PD-L1 axis plays a critical role in allowing tumors to evade T cell immunological responses [4]. The use of PD-1 inhibitors or PD-L1 inhibitors to sensitize tumor therapy can reverse this evasion, enhancing anti-tumor immune activity, and the FDA has approved PD-L1 inhibitors such as Atezolizumab, Durvalumab, and Avelumab for use in cancer therapy [4]. Cytotoxic T lymphocytes are primarily responsible for killing tumor cells [5], and they play an important role in effective anti-PD-1/PD-L1 therapy within the tumor microenvironment [6]. Disrupting T cell exhaustion through the PD-1/PD-L1 axis has impressive therapeutic efficacy in the treatment of melanoma [7].

Oxidative stress significantly impacts melanogenesis and subsequent melanoma formation [8]. While melanin plays a protective role against the progression of cutaneous carcinomas such as melanoma, the presence of melanin could be required for the malignant transformation of melanocytes [9]. Melanogenesis can promote tumor growth and induce tumor progression [10,11]. Melanogenesis is enhanced by the activation of tyrosinase, an essential enzyme in melanin synthesis, and is initiated by the oxidation of tyrosine by tyrosinase [12].

SH003, a combination of natural products derived from *Astragalus membranaceus* (Fisch.) Bunge, *Angelica gigas* Nakai, and *Trichosanthes kirilowii* (Maxim.), has been commonly used in East Asia. Studies have revealed that SH003 possesses anti-tumor effects in several cancer types such as breast [13], lung [14], gastric [15], cervical [16], and prostate cancer [17], as well as anti-oxidant effects [18]. Additionally, SH003 has shown beneficial effects in alleviating neuropathic pain induced by anti-cancer agents [19,20]. Formononetin (FMN), which is a constituent of *Astragalus membranaceus* (Fisch.) Bunge [21], has been reported to be a marker compound (0.06%) of SH003 [22,23,24]. FMN has been recognized for its beneficial properties in combatting several cancers, including esophageal [21], colon [25], and breast cancer [26], and in mitigating oxidative damage, infection, inflammation, diabetes, and obesity [27,28]. Regarding the production of melanin, there is only one piece of research that suggests FMN has an anti-oxidant effect by inhibiting tyrosinase activity in HepG2 cells [29]. However, there has been no research on the regulatory effect of SH003 and FMN on melanogenesis and melanoma.

Accordingly, we aimed to identify the anti-melanogenesis and anti-cancer activity of SH003 and FMN with emphasis on the immune checkpoint in the melanoma microenvironment. We investigated the potential of SH003 and FMN as anti-melanogenesis inhibitors and anti-cancer agents for combatting melanoma using B16F10 cells. Furthermore, the cytotoxic efficacy of cytotoxic T lymphocytes by SH003 and FMN was evaluated using CTLL-2 cells.

## 2. Materials and Methods

### 2.1. SH003 and FMN

SH003 was extracted from *Astragalus membranaceus* (Fisch.) Bunge, *Angelica gigas* Nakai, and *Trichosanthes kirilowii* (Maxim.) in a 1:1:1 ratio as previously described [15]. FMN (purity ≥ 99%; Sigma-Aldrich Co., Burlington, MA, USA) was prepared in 0.01% dimethyl sulfoxide.

### 2.2. Cell Lines

B16F10 cells (Korean Cell Line Bank, Seoul, Republic of Korea) and CTLL-2 cells (American Type Culture Collection, Manassas, VA, USA) were cultured according to the manufacturer’s instructions. B16F10 cells were cultured in Dulbecco’s Modified Eagle’s Medium with 10% fetal bovine serum. CTLL-2 cells were cultured in RPMI 1640 medium containing 10% T-cell culture supplement with Con A (T-STIM with Con A, BD Biosciences, San Jose, CA, USA) and 10% fetal bovine serum.

### 2.3. Melanin Content and Tyrosinase Activity Assay

The intracellular melanin content and tyrosinase activity were assessed with reference to previous works [30,31,32]. B16F10 cells (1 × 10^5^ cells) were treated with SH003, FMN, and α-melanocyte-stimulating hormone (α-MSH, 100 nM) for 72 h. For the melanin content assay, the cells were washed with phosphate-buffered saline and the resulting pellets were lysed with 150 μL of 1 N NaOH containing 10% dimethyl sulfoxide for 1 h at 80 °C. The cell suspension was quantified by means of a bicinchoninic acid protein assay kit (Thermo Fisher Scientific, Waltham, MA, USA). The melanin content in the quantified cell suspension was assessed by means of a colorimetric microplate reader (405 nm, Molecular Devices, San Jose, CA, USA). In the tyrosinase activity assay, the cells were lysed in 1% Triton X-100 for 2 h at 4 °C. The quantified pellets were then added to 10 μL of 10 mM L-DOPA (also known as levodopa) for 30 min at 37 °C. Tyrosinase activity was assessed by means of a microplate reader (475 nm, Molecular Devices). The melanin content and tyrosinase activity were calculated as 100% of the α-MSH-treated group.

### 2.4. MTT Assay

Cell viability was assessed by means of a 3-(4,5-dimethylthiazol-2-yl)-2,5-diphenyltetrazolium Bromide (MTT) assay referring to existing work [18]. The absorbance was evaluated spectrophotometrically by means of a colorimetric microplate reader (570 nm, Molecular Devices), after the formazan crystals were dissolved in dimethyl sulfoxide.

### 2.5. Western Blot Analysis

Each expression was detected with reference to existing work [18]. The harvested cells were extracted by means of radioimmunoprecipitation assay buffer (Sigma-Aldrich Co.) and then quantified by means of a bicinchoninic acid protein assay kit (Thermo Fisher Scientific). The total protein was separated by sodium dodecyl sulfate-polyacrylamide gel electrophoresis and transferred to nitrocellulose membranes (Bio-Rad Laboratories, Hercules, CA, USA). The membranes were incubated with primary antibodies (PARP, caspase-3, PD-L1, phosphorylated signal transducer and activator of transcription 1 (pSTAT1), STAT1, actin, and GAPDH; Santa Cruz Biotechnology, Inc., Dallas, TX, USA). An enhanced chemiluminescence reagent (DoGenBio, Seoul, Republic of Korea) was used for signal development.

### 2.6. Cell Cycle Analysis

Cell cycle phase and DNA content were assessed using a propidium iodide staining and flow cytometry assay with a propidium iodide flow cytometry kit (abcam, Boston, MA, USA). The collected cells were fixed with 70% ice-cold ethanol and stored overnight at −20 °C. After washing with phosphate-buffered saline, the pellets were then re-suspended in phosphate-buffered saline containing 50 μg/mL of propidium iodide and 100 μg/mL of ribonuclease and incubated for 30 min at room temperature. The cells were then subjected to analysis using a FACSCalibur flow cytometer (BD Biosciences).

### 2.7. Cell Apoptosis Assay

Apoptotic cells were measured using a FITC Annexin V apoptosis detection kit with propidium iodide (Biolegend, San Diego, CA, USA). The collected cells for apoptosis analysis were double-stained with FITC-conjugated Annexin V and propidium iodide in a binding buffer for 30 min. The analysis was performed using a FACSCalibur flow cytometer.

### 2.8. qPCR

Total RNA was prepared by means of an extraction kit (iNtRON Biotech Inc., Seongnam, Republic of Korea). Reverse transcription and qPCR were performed using a cDNA synthesis kit (Bioneer, Inc., Daejeon, Republic of Korea) and Power SYBR^®^ Green Master Mix (Thermo Fisher Scientific) on a Real-Time PCR System (Applied Biosystems, Waltham, MA, USA). Primers for genes (PD-L1, For: 5′-TGCTGCATAATCAGCTACGG-3′, Rev: 5′-GCTGGTCACATTGAGAAGCA-3′ and GAPDH, For: 5′-CCAATGTGTCCGTCGTGGATCT-3′, Rev: 5′- GTTGAAGTCGCAGGAGACAACC-3′) were synthesized at Bioneer, Inc.

### 2.9. Immunofluorescent Staining

Immunofluorescent staining was conducted as previously described [18] with anti-PD-L1 (Santa Cruz Biotechnology) and anti-mouse Alexa Fluor^®^ 647 secondary antibodies (abcam).

### 2.10. T Cell-Mediated Cytotoxicity Assay

To determine the cytotoxic T lymphocyte activities of CTLL-2 cells, a cell-mediated cytotoxicity assay was conducted using a cell cytotoxicity assay kit (DoGenBio). CTLL-2 cells (effector cells) were cultured with B16F10 cells (target cells) at an effector-to-target cell ratio of 20:1. The cells were exposed to SH003 and FMN for 24 h. The lactate dehydrogenase release was analyzed in the cell-free supernatants. Additionally, a viability assay was performed using the MTT assay on the adhered viable target cells.

### 2.11. Statistical Analysis

Data are shown as the mean ± standard error of the mean (SEM). Multiple comparisons were conducted using one-way analysis of variance followed by Tukey’s *post hoc* test. A Student’s unpaired *t*-test was used to compare between two independent groups. A *p*-value below 0.05 was considered statistically significant (IBM SPSS software, Armonk, NY, USA).

## 3. Results

### 3.1. Regulatory Effect of SH003 and FMN on the Melanogenesis of B16F10 Cells

**Figure 1:**
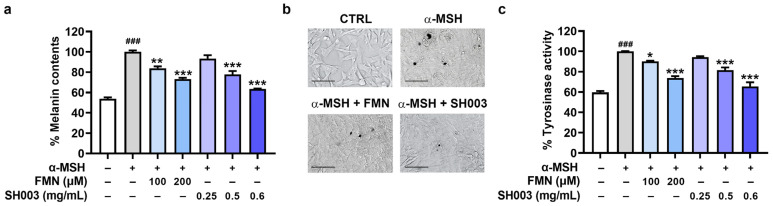
SH003 and FMN reduce melanin content. (**a**) The melanin contents and (**c**) tyrosinase activity were assessed in α-MSH-stimulated B16F10 cells (n = 4 per group). (**b**) The dark pigmentation was assessed under a light microscope. The scale bar represents 100 μm. ^###^ *p* < 0.001 vs. control (CTRL, untreated) group. * *p* < 0.05, ** *p* < 0.01, and *** *p* < 0.001 vs. α-MSH-treated group.

First, we assessed the effects of SH003 and FMN on melanin content in α-MSH-stimulated B16F10 cells. α-MSH is one of the most common reagents used to stimulate pigmentation [33]. The α-MSH stimulation augmented the melanin content in B16F10 cells. However, treatment with SH003 and FMN significantly reduced the α-MSH-induced increase in melanin content (*p* < 0.05, Figure 1a). In addition, the cell images in Figure 1b show that SH003 and FMN clearly attenuated the presence of dark pigments. Next, we investigated whether SH003 and FMN could regulate tyrosinase activity in the α-MSH-stimulated B16F10 cells. As expected, SH003 and FMN significantly inhibited tyrosinase activity (*p* < 0.05, Figure 1c).

### 3.2. Regulatory Effect of SH003 and FMN on the Proliferation and Apoptosis of B16F10 Cells

**Figure 2:**
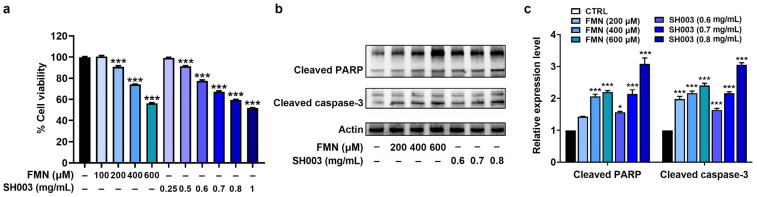
SH003 and FMN inhibit B16F10 cell growth. (**a**) Proliferation activity of B16F10 cells was assessed using an MTT assay after incubation with SH003 and FMN for 48 h (n = 8 per group). (**b**) PARP and caspase-3 levels were detected using immunoblots after incubation with SH003 and FMN for 24 h. (**c**) Expression levels were quantified relative to the CTRL group. * *p* < 0.05 and *** *p* < 0.001 vs. control (CTRL, untreated) group.

To assess the anti-cancer effect of SH003 and FMN, an MTT assay was first performed at different concentrations. As illustrated in Figure 2a, cell viability gradually decreased with increasing concentrations of SH003 and FMN, indicating that SH003 and FMN had inhibitory effects on the proliferation of B16F10 cells (*p* < 0.05). To further explore the molecular mechanism of this effect, we investigated the expression of signaling molecules by immunoblots. Both SH003 and FMN significantly enhanced the levels of cleaved PARP and caspase-3 compared with the control group in B16F10 cells, (*p* < 0.05, Figure 2b,c).

### 3.3. Synergistic Effect of Cisplatin Combined with SH003 and FMN on Cell Cycle Arrest of B16F10 Cells

**Figure 3:**
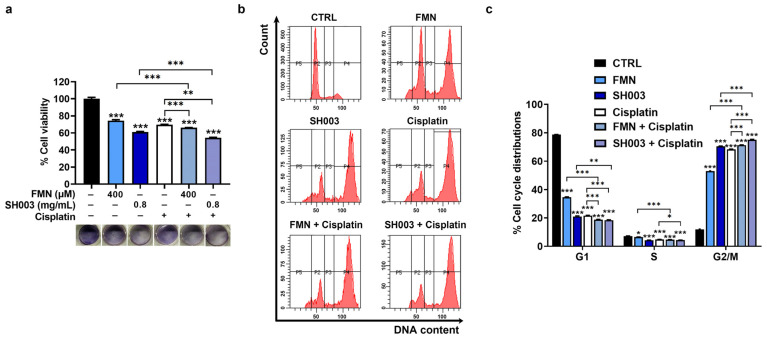
The combination of cisplatin with SH003 and FMN has a synergistic effect on cell cycle arrest. (**a**) The cell viability of B16F10 cells was analyzed with an MTT assay after incubation with SH003, FMN, and cisplatin (20 µM) for 48 h (n = 8 per group). (**b**,**c**) Cell cycle distribution was assessed using flow cytometry (n = 4 per group). * *p* < 0.05, ** *p* < 0.01, and *** *p* < 0.001 vs. control (CTRL, untreated) group.

It has been known that cisplatin is a good chemotherapy drug for the treatment of various tumors [34]. To verify if combining cisplatin with SH003 and FMN inhibits tumor progression more effectively, we examined cell viability in the presence of SH003 and FMN. Based on the IC 50 values (SH003, ≈0.78 mg/mL; FMN, ≈468.6 μM), we selected concentrations of 0.8 mg/mL for SH003 and 400 µM for FMN for subsequent experiments. The MTT assay indicated that the combination of cisplatin with SH003 and the combination of cisplatin with FMN exhibited significantly lower viability compared with each agent alone (*p* < 0.05, Figure 3a). Furthermore, analysis of the cell cycle distribution of SH003 and FMN showed that both SH003 and FMN led to cell cycle arrest at the G2/M phase (*p* < 0.05, Figure 3b,c). In addition, the effect of the combination groups on the cell cycle arrest was stronger than each single agent group (*p* < 0.05).

### 3.4. Synergistic Effect of Cisplatin Combined with SH003 and FMN on Apoptosis of B16F10 Cells

**Figure 4:**
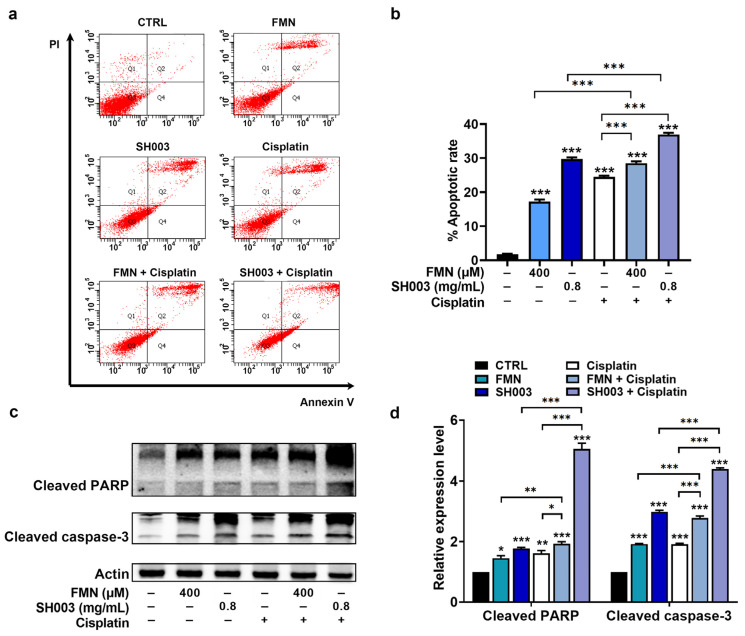
The combination of cisplatin with SH003 and FMN has a synergistic effect on apoptosis. (**a**,**b**) B16F10 cells were treated with SH003, FMN, and cisplatin (20 µM) for 48 h. The percent of apoptosis cells was assessed using flow cytometry (n = 4 per group). (**c**) PARP and caspase-3 levels were detected using immunoblots after incubation with SH003, FMN, and cisplatin for 24 h. (**d**) Expression levels were quantified relative to the CTRL group. * *p* < 0.05, ** *p* < 0.01, and *** *p* < 0.001 vs. control (CTRL, untreated) group.

To assess the effect of SH003 and FMN on apoptosis in B16F10 cells, a cell apoptosis assay was carried out. As expected, SH003 and FMN significantly augmented the apoptosis of B16F10 cells compared with the control group (*p* < 0.05, Figure 4a,b). Furthermore, the combination groups of cisplatin with SH003 and FMN showed a higher apoptosis rate than each agent alone (*p* < 0.05, Figure 4a,b). In addition, immunoblot analysis indicated elevated levels of cleaved PARP and cleaved caspase-3 levels in both the SH003-treated and FMN-treated groups compared to the control group. The combination groups also demonstrated remarkable elevation in these levels (*p* < 0.05, Figure 4c,d).

### 3.5. Regulatory Effect of SH003 and FMN on IFN-γ-induced PD-L1 Expression in B16F10 Cells

**Figure 5:**
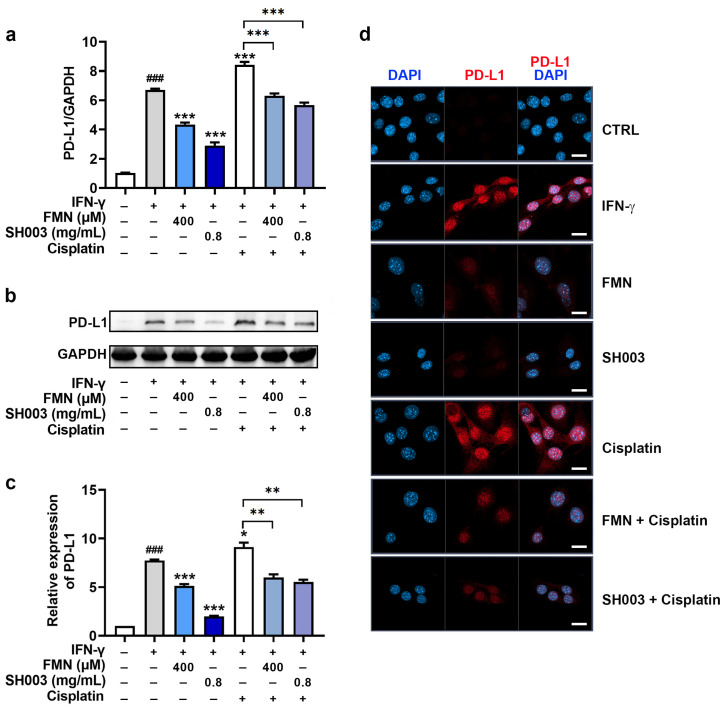
SH003 and FMN inhibit IFN-γ-induced PD-L1 expression. (**a**) The mRNA levels (n = 5 per group) and (**b**) protein levels of PD-L1 were assessed by qPCR and immunoblots after incubation with SH003, FMN, and cisplatin (20 μM) for 1 h and further incubation with IFN-γ (10 ng/mL) for 24 h. (**c**) Each expression was quantified relative to the CTRL group. ^###^ *p* < 0.001 vs. control (CTRL, untreated) group. * *p* < 0.05, ** *p* < 0.01, and *** *p* < 0.001 vs. IFN-γ-treated group. (**d**) Representative confocal microscopy images for PD-L1. The scale bar represents 20 µm.

**Figure 6:**
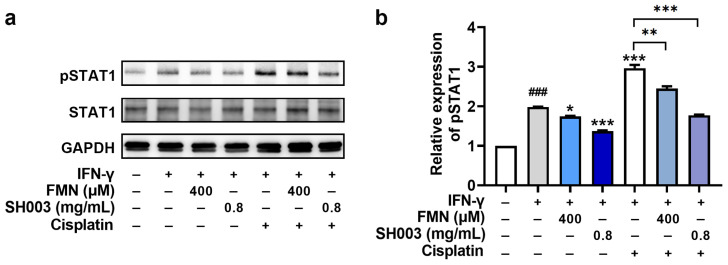
SH003 and FMN reduce IFN-γ-induced STAT1 phosphorylation. (**a**) The protein levels of pSTAT1 were detected using immunoblots after incubation with SH003, FMN, and cisplatin (20 μM) for 1 h and further incubation with IFN-γ (10 ng/mL) for 10 min. (**b**) Expression levels were quantified relative to the CTRL group. ^###^ *p* < 0.001 vs. control (CTRL, untreated) group. * *p* < 0.05, ** *p* < 0.01, and *** *p* < 0.001 vs. IFN-γ-treated group.

IFN-γ is involved in tumor immune evasion by stimulating the expression of PD-L1 on tumor cells, suppressing the effector functions of cytotoxic T lymphocytes, and helping tumor cells evade immune destruction [35]. Thus, we investigated whether SH003 and FMN could affect IFN-γ-induced PD-L1 expression in B16F10 cells. PCR analysis showed that SH003 and FMN significantly reduced the IFN-γ-induced PD-L1 mRNA expression levels (*p* < 0.05, Figure 5a). Additionally, SH003 and FMN effectively inhibited the increase in PD-L1 protein expression induced by IFN-γ (*p* < 0.05, Figure 5b,c). Images of PD-L1 staining further show the decrease in PD-L1 expression following SH003 and FMN treatment (Figure 5d). Interestingly, cisplatin has been reported to cause tumor immune evasion by up-regulating PD-L1 [36,37]. As illustrated in Figure 5, cisplatin enhanced IFN-γ-induced PD-L1 expression levels. However, SH003 and FMN significantly reduced the increase in IFN-γ-induced PD-L1 expression caused by cisplatin (*p* < 0.05). Subsequently, the mechanism for the modulation of PD-L1 expression was further explored. Figure 6 shows that SH003 and FMN significantly decreased the IFN-γ-induced phosphorylation of STAT1, which was enhanced by cisplatin (*p* < 0.05).

### 3.6. Regulatory Effect of SH003 and FMN on Cytotoxicity of CTLL-2 Cells against B16F10 Cells

**Figure 7:**
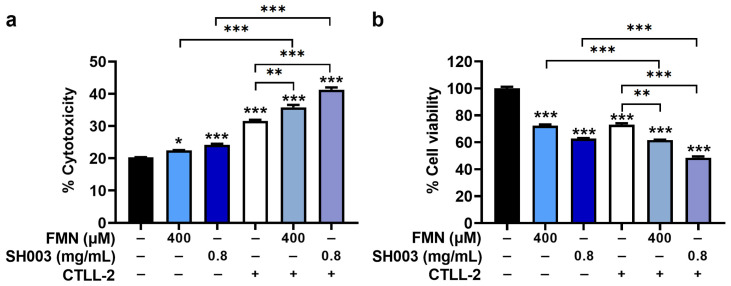
SH003 and FMN enhance the cytotoxicity of CTLL-2 cells against B16F10 cells. (**a**) Cytotoxicity was determined using a lactate dehydrogenase detection assay in a co-culture system of B16F10 cells/CTLL-2 cells (n = 4 per group). (**b**) The cell viability was determined with an MTT assay in a co-culture system of B16F10/CTLL-2 cells (n = 4 per group). * *p* < 0.05, ** *p* < 0.01, and *** *p* < 0.001 vs. control (untreated) group.

To further assess the anti-cancer effect of SH003 and FMN, the regulatory effect on the cytotoxicity of cytotoxic T lymphocytes was evaluated using CTLL-2 cells. As demonstrated in Figure 7a, CTLL-2 cells showed cytotoxicity against B16F10 cells, and SH003 and FMN significantly augmented the cytotoxicity of CTLL-2 cells against B16F10 cells (*p* < 0.05). Subsequently, CTLL-2 cells downregulated the cell viability of B16F10 cells, and SH003 and FMN further suppressed the cell viability of B16F10 cells decreased by CTLL-2 cells (*p* < 0.05, Figure 7b).

## 4. Discussion

Carcinogenesis is strongly associated with oxidative stress and nutritional status [38]. The nutritional status affects the immune system, which in turn can impact cancer progression [38,39]. Cancer initiation is also regulated by nutrition-mediated oxidation, which can stimulate oncogenic pathways [38,39]. *Astragalus membranaceus* (Fisch.) Bunge, *Angelica gigas* Nakai, and *Trichosanthes kirilowii* (Maxim.), as the functional foods that comprise SH003, possess not only high nutritional value but also exhibit anti-cancer and anti-oxidant properties [22,40,41,42,43,44]. Our previous work also showed the immune-enhancing effects of SH003 [18]. Thus, the immune-enhancing and anti-oxidant activities of SH003 strongly support the potential of SH003 as an anti-cancer agent. However, more experiments involving the efficacy of SH003 against nutrition-mediated oxidation in various tumor models are needed to verify this potential.

The variations were observed in the anti-melanogenic and anti-cancer effects between SH003 and FMN in our work. Calycosin, an isoflavone of *Astragalus membranaceus* (Fisch.) Bunge, has been reported to have anti-melanogenesis effects in an animal model [45]. Decursin, an effective compound of *Angelica gigas* Nakai, has been reported to possess protective effects against hyperpigmentation by inhibiting melanin synthesis and tyrosinase activity of B16F10 melanoma cells [46]. Decursin also exerted beneficial properties against melanoma by reducing B16F10 melanoma growth via induction of apoptosis [47]. Nodakenin, an effective compound of *Angelica gigas* Nakai, showed anti-melanogenesis effects by decreasing the melanin synthesis and tyrosinase activity of B16F10 melanoma cells [48]. Oh et al. [49] suggested cucurbitacin, an effective compound of *Trichosanthes kirilowii* (Maxim.), as a regulatory component on melanin synthesis and tyrosinase activity of B16F10 melanoma cells. Tsao et al. [50] revealed that trichosanthin, an effective compound of *Trichosanthes kirilowii* (Maxim.), displayed cytotoxic activity against melanoma cells. Thus, the variations might be observed because SH003, being a mixture of *Astragalus membranaceus* (Fisch.) Bunge, *Angelica gigas* Nakai, and *Trichosanthes kirilowii* (Maxim.), contains a range of active compounds such as calycosin, decursin, nodakenin, cucurbitacins, and trichosanthin, whereas FMN is just one of the active compounds in SH003.

Melanin plays a critical role in melanoma pathology by promoting malignant transformation of melanocytes [51]. The melanin presence in melanoma cells can impact their susceptibility to treatment [52]. Slominski et al. [53] suggested that a decrease in melanogenesis could form a therapeutic approach in the management of advanced melanoma. In fact, melanogenesis inhibition improved the outcome of radiotherapy in metastatic melanoma patients [52]. Moreover, oxidative stress is closely related to melanogenesis [8]. Antioxidants derived from natural products have been found to inhibit melanin content in B16 cells [54]. In our work, we revealed that SH003 reduced melanin content and tyrosine oxidation in B16F10 cells, proposing its potential as an antioxidant, at least in this respect. Thus, this suggests that SH003 affects tumor progression by modulating melanin synthesis. However, the function of melanin in melanoma is controversial. A study suggested that melanin inhibits melanoma metastasis [55]. Therefore, further studies are required to examine the regulatory effect of SH003 on melanogenesis using various melanoma models.

PD-L1 expression in tumor cells correlates with tumor immune evasion and poor clinical outcomes [56]. Accumulating evidence has shown the anti-tumor effects of suppressing PD-L1 expression in tumor cells [57,58]. In our study, SH003 effectively blocked the progression of melanoma by targeting the PD-L1 signaling pathway and promoting cell apoptosis. PD-L1 is known to induce DNA damage repair and inhibit cancer cell death in response to DNA damage [59]. Thus, the advantages of using SH003 to sensitize tumor therapy via the modulation of PD-L1 may also be mediated by PD-L1-induced DNA damage repair. However, further research is needed to provide evidence for the advantages of SH003 against PD-L1-induced DNA damage. The binding of PD-L1 to PD-1 disrupts the ability of T cells to kill tumor cells [57]. PD-1-expressing CTLL-2 cells have been used as a murine cell line of cytotoxic T lymphocytes [60,61]. In addition, the co-culture system of CTLL-2 and B16F10 cells has been applied in several studies to evaluate anti-cancer efficacy, including T cell-mediated melanoma killing [62,63]. In our work, SH003 augmented the killing effect of CTLL-2 cells on B16F10 cells by reducing PD-L1 via the downregulation of STAT1 signaling. These findings suggest that SH003 could be a potential immuno-anti-cancer agent for the treatment of melanoma. However, further research is required to demonstrate the ability of SH003 to enhance T cell immunity by modulating PD-L1 levels in various tumor-associated antigen-presenting cells.

Cisplatin augments PD-L1 expression in tumor cells [36,37]. Thus, combination therapy involving cisplatin and immune checkpoint inhibitors has been proposed as a strategy to address this [64]. In our study, SH003 demonstrated significant anti-cancer potential against melanoma by reducing tumor proliferation and promoting apoptosis. The combination of SH003 with cisplatin exhibited even higher anti-cancer activity compared to cisplatin alone. This enhancement can be attributed to an increased number of apoptotic cells and elevated expression of PARP and caspase-3 in the combination group. The combination also showed a more pronounced cell cycle arrest, leading to an elevated proportion of cells in the G2/M phase. These results suggest that SH003 could be a valuable agent for combination therapy with cisplatin, effectively overcoming PD-L1-mediated resistance to chemotherapy and immunotherapy.

## 5. Conclusions

The current work is the first to characterize the anti-melanogenesis and anti-melanoma properties of SH003 and FMN. The principal findings of this study are as follows: (i) SH003 and FMN treatments inhibit melanogenesis. (ii) SH003 and FMN promote apoptosis in B16F10 cells, accompanied by elevated levels of active PARP and caspase-3. (iii) The combination of cisplatin with SH003 or FMN enhances their respective pro-apoptotic effects. (iv) SH003 and FMN reduce PD-L1 expression by downregulating STAT1 phosphorylation. (v) SH003 and FMN augment the cytotoxicity of cytotoxic T lymphocytes against B16F10 cells. Therefore, SH003 has anti-melanoma properties, simultaneously enhancing the function of cytotoxic T lymphocytes. These results propose that the natural product mixture SH003 holds promise as a novel approach in the prevention of melanoma progression. However, future studies should explore the comparative advantages of SH003 and FMN in clinical application.

## Data Availability

The data sets used and/or analyzed during the current study are available from the corresponding author.

## References

[1] Sample A., He Y.Y. (2018). Mechanisms and prevention of UV-induced melanoma. Photodermatol. Photoimmunol. Photomed..

[2] Sung H., Ferlay J., Siegel R.L., Laversanne M., Soerjomataram I., Jemal A., Bray F. (2021). Global Cancer Statistics 2020: GLOBOCAN Estimates of Incidence and Mortality Worldwide for 36 Cancers in 185 Countries. CA Cancer J. Clin..

[3] Dermani F.K., Samadi P., Rahmani G., Kohlan A.K., Najafi R. (2019). PD-1/PD-L1 immune checkpoint: Potential target for cancer therapy. J. Cell. Physiol..

[4] Gong J., Chehrazi-Raffle A., Reddi S., Salgia R. (2018). Development of PD-1 and PD-L1 inhibitors as a form of cancer immunotherapy: A comprehensive review of registration trials and future considerations. J. Immunother. Cancer.

[5] Thomas D.A., Massagué J. (2005). TGF-beta directly targets cytotoxic T cell functions during tumor evasion of immune surveillance. Cancer Cell.

[6] Wang X., Zha H., Wu W., Yuan T., Xie S., Jin Z., Long H., Yang F., Wang Z., Zhang A. (2023). CD200+ cytotoxic T lymphocytes in the tumor microenvironment are crucial for efficacious anti-PD-1/PD-L1 therapy. Sci. Transl. Med..

[7] Greil R., Hutterer E., Hartmann T.N., Pleyer L. (2017). Reactivation of dormant anti-tumor immunity—A clinical perspective of therapeutic immune checkpoint modulation. Cell Commun. Signal..

[8] Kamiński K., Kazimierczak U., Kolenda T. (2022). Oxidative stress in melanogenesis and melanoma development. Contemp. Oncol..

[9] Slominski R.M., Sarna T., Płonka P.M., Raman C., Brożyna A.A., Slominski A.T. (2022). Melanoma, Melanin, and Melanogenesis: The Yin and Yang Relationship. Front. Oncol..

[10] Slominski R.M., Zmijewski M.A., Slominski A.T. (2015). The role of melanin pigment in melanoma. Exp. Dermatol..

[11] Moan J., Dahlback A., Setlow R.B. (1999). Epidemiological support for an hypothesis for melanoma induction indicating a role for UVA radiation. Photochem. Photobiol..

[12] Gillbro J.M., Olsson M.J. (2011). The melanogenesis and mechanisms of skin-lightening agents--existing and new approaches. Int. J. Cosmet. Sci..

[13] Lee S.Y., Kim T.H., Choi W.G., Chung Y.H., Ko S.G., Cheon C., Cho S.G. (2023). SH003 Causes ER Stress-mediated Apoptosis of Breast Cancer Cells via Intracellular ROS Production. Cancer Genom. Proteom..

[14] Jeong M.S., Lee K.W., Choi Y.J., Kim Y.G., Hwang H.H., Lee S.Y., Jung S.E., Park S.A., Lee J.H., Joo Y.J. (2021). Synergistic Antitumor Activity of SH003 and Docetaxel via EGFR Signaling Inhibition in Non-Small Cell Lung Cancer. Int. J. Mol. Sci..

[15] Kim T.W., Cheon C., Ko S.G. (2020). SH003 activates autophagic cell death by activating ATF4 and inhibiting G9a under hypoxia in gastric cancer cells. Cell Death Dis..

[16] Lee K.M., Lee K., Choi Y.K., Choi Y.J., Seo H.S., Ko S.G. (2017). SH003-induced G1 phase cell cycle arrest induces apoptosis in HeLa cervical cancer cells. Mol. Med. Rep..

[17] Choi Y.J., Choi Y.K., Lee K.M., Cho S.G., Kang S.Y., Ko S.G. (2016). SH003 induces apoptosis of DU145 prostate cancer cells by inhibiting ERK-involved pathway. BMC Complement. Altern. Med..

[18] Han N.R., Kim K.C., Kim J.S., Ko S.G., Park H.J., Moon P.D. (2022). The immune-enhancing effects of a mixture of Astragalus membranaceus (Fisch.) Bunge, Angelica gigas Nakai, and Trichosanthes Kirilowii (Maxim.) or its active constituent nodakenin. J. Ethnopharmacol..

[19] Lee J.H., Kim B., Ko S.G., Kim W. (2022). Analgesic Effect of SH003 and Trichosanthes kirilowii Maximowicz in Paclitaxel-Induced Neuropathic Pain in Mice. Curr. Issues Mol. Biol..

[20] Lee K., Ku J.M., Choi Y.J., Hwang H.H., Jeong M., Kim Y.G., Kim M.J., Ko S.G. (2021). Herbal Prescription SH003 Alleviates Docetaxel-Induced Neuropathic Pain in C57BL/6 Mice. Evid. Based Complement. Alternat. Med..

[21] Chen L., Xing D., Guo L.R., Jin J., Li S. (2023). Formononetin, an Active Component of Astragalus Membranaceus, Inhibits the Pathogenesis and Progression of Esophageal Cancer Through the COX-2/Cyclin D1 Axis. Clin. Lab..

[22] Han N.R., Park H.J., Ko S.G., Moon P.D. (2023). The Protective Effect of a Functional Food Consisting of Astragalus membranaceus, Trichosanthes kirilowii, and Angelica gigas or Its Active Component Formononetin against Inflammatory Skin Disorders through Suppression of TSLP via MDM2/HIF1α Signaling Pathways. Foods.

[23] Choi Y.K., Cho S.G., Woo S.M., Yun Y.J., Park S., Shin Y.C., Ko S.G. (2014). Herbal extract SH003 suppresses tumor growth and metastasis of MDA-MB-231 breast cancer cells by inhibiting STAT3-IL-6 signaling. Mediat. Inflamm..

[24] Choi Y.J., Choi W.G., Lee K., Jeong M., Park S.C., Jang Y.P., Ko S.G. (2022). The effect of isoflavonoid contents in SH003 and its subfractions on breast cancer. J. Korean Med..

[25] Hu Y., Zhai W., Tan D., Chen H., Zhang G., Tan X., Zheng Y., Gao W., Wei Y., Wu J. (2023). Uncovering the effects and molecular mechanism of Astragalus membranaceus (Fisch.) Bunge and its bioactive ingredients formononetin and calycosin against colon cancer: An integrated approach based on network pharmacology analysis coupled with experimental validation and molecular docking. Front. Pharmacol..

[26] Song X., Li J. (2022). Screening of Immune-Related Genes and Predicting the Immunotherapeutic Effects of Formononetin in Breast Cancer: A Bioinformatics Analysis. Evid. Based Complement. Altern. Med..

[27] Mu H., Bai Y.H., Wang S.T., Zhu Z.M., Zhang Y.W. (2009). Research on antioxidant effects and estrogenic effect of formononetin from Trifolium pratense (red clover). Phytomedicine.

[28] Machado Dutra J., Espitia P.J.P., Andrade Batista R. (2021). Formononetin: Biological effects and uses—A review. Food Chem..

[29] Kim J.H., Cho I.S., So Y.K., Kim H.H., Kim Y.H. (2018). Kushenol A and 8-prenylkaempferol, tyrosinase inhibitors, derived from Sophora flavescens. J. Enzyme Inhib. Med. Chem..

[30] Zhou S., Riadh D., Sakamoto K. (2021). Grape Extract Promoted α-MSH-Induced Melanogenesis in B16F10 Melanoma Cells, Which Was Inverse to Resveratrol. Molecules.

[31] Bayrakçeken Güven Z., Saracoglu I., Nagatsu A., Yilmaz M.A., Basaran A.A. (2023). Anti-tyrosinase and antimelanogenic effect of cinnamic acid derivatives from Prunus mahaleb L.: Phenolic composition, isolation, identification and inhibitory activity. J. Ethnopharmacol..

[32] Chen Y.S., Lee S.M., Lin C.C., Liu C.Y. (2014). Hispolon decreases melanin production and induces apoptosis in melanoma cells through the downregulation of tyrosinase and microphthalmia-associated transcription factor (MITF) expressions and the activation of caspase-3, -8 and -9. Int. J. Mol. Sci..

[33] Chung S., Lim G.J., Lee J.Y. (2019). Quantitative analysis of melanin content in a three-dimensional melanoma cell culture. Sci. Rep..

[34] Dasari S., Tchounwou P.B. (2014). Cisplatin in cancer therapy: Molecular mechanisms of action. Eur. J. Pharmacol..

[35] Burr M.L., Sparbier C.E., Chan Y.C., Williamson J.C., Woods K., Beavis P.A., Lam E.Y.N., Henderson M.A., Bell C.C., Stolzenburg S. (2017). CMTM6 maintains the expression of PD-L1 and regulates anti-tumour immunity. Nature.

[36] Wangpaichitr M., Kandemir H., Li Y.Y., Wu C., Nguyen D., Feun L.G., Kuo M.T., Savaraj N. (2017). Relationship of Metabolic Alterations and PD-L1 Expression in Cisplatin Resistant Lung Cancer. Cell Dev. Biol..

[37] Tran L., Allen C.T., Xiao R., Moore E., Davis R., Park S.J., Spielbauer K., Van Waes C., Schmitt N.C. (2017). Cisplatin Alters Antitumor Immunity and Synergizes with PD-1/PD-L1 Inhibition in Head and Neck Squamous Cell Carcinoma. Cancer Immunol. Res..

[38] Saha S.K., Lee S.B., Won J., Choi H.Y., Kim K., Yang G.M., Dayem A.A., Cho S.G. (2017). Correlation between Oxidative Stress, Nutrition, and Cancer Initiation. Int. J. Mol. Sci..

[39] Valdés-Ramos R., Benítez-Arciniega A.D. (2007). Nutrition and immunity in cancer. Br. J. Nutr..

[40] Che D., Adams S., Wei C., Gui-Xin Q., Atiba E.M., Hailong J. (2019). Effects of Astragalus membranaceus fiber on growth performance, nutrient digestibility, microbial composition, VFA production, gut pH, and immunity of weaned pigs. Microbiol. Open.

[41] Kim D.S., Kim H.S., Lee J., Hong S.J., Cho J.J., Cho K.M., Shin E.C. (2019). Comprehensive changes in volatile/nonvolatile compounds and flavor and physicochemical characteristics in Angelica gigas Nakai roots by thermal processing. J. Food Biochem..

[42] Xu L., Zhu C., Liu T., Karrar E., Ouyang Y., Li D. (2022). Effect of microwave heating on lipid composition, chemical properties and antioxidant activity of oils from Trichosanthes kirilowii seed. Food Res. Int..

[43] Zhang H.Q., Liu P., Duan J.A., Dong L., Shang E.X., Qian D.W., Zhu Z.H., Li H.W., Li W.W. (2019). Comparative Analysis of Carbohydrates, Nucleosides and Amino Acids in Different Parts of Trichosanthes kirilowii Maxim. by (Ultra) High-Performance Liquid Chromatography Coupled with Tandem Mass Spectrometry and Evaporative Light Scattering Detector Methods. Molecules.

[44] Lee K., Youn B.Y., Choi Y.J., Moon S., Im J., Cho K., Ko S.G., Cheon C. (2022). State of the Art and Future Implications of SH003: Acting as a Therapeutic Anticancer Agent. Cancers.

[45] Tayier N., Qin N.Y., Zhao L.N., Zeng Y., Wang Y., Hu G., Wang Y.Q. (2021). Theoretical Exploring of a Molecular Mechanism for Melanin Inhibitory Activity of Calycosin in Zebrafish. Molecules.

[46] Choi H., Yoon J.H., Youn K., Jun M. (2022). Decursin prevents melanogenesis by suppressing MITF expression through the regulation of PKA/CREB, MAPKs, and PI3K/Akt/GSK-3β cascades. Biomed. Pharmacother..

[47] Kim B.S., Seo H., Kim H.J., Bae S.M., Son H.N., Lee Y.J., Ryu S., Park R.W., Nam J.O. (2015). Decursin from Angelica gigas Nakai Inhibits B16F10 Melanoma Growth Through Induction of Apoptosis. J. Med. Food.

[48] Yoon Y., Bae S., Kim T.J., An S., Lee J.H. (2023). Nodakenin Inhibits Melanogenesis Via the ERK/MSK1 Signaling Pathway. Pharmazie.

[49] Oh H., Mun Y.J., Im S.J., Lee S.Y., Song H.J., Lee H.S., Woo W.H. (2002). Cucurbitacins from Trichosanthes kirilowii as the inhibitory components on tyrosinase activity and melanin synthesis of B16/F10 melanoma cells. Planta Med..

[50] Tsao S.W., Yan K.T., Yeung H.W. (1986). Selective killing of choriocarcinoma cells in vitro by trichosanthin, a plant protein purified from root tubers of the Chinese medicinal herb Trichosanthes kirilowii. Toxicon.

[51] Cabaço L.C., Tomás A., Pojo M., Barral D.C. (2022). The Dark Side of Melanin Secretion in Cutaneous Melanoma Aggressiveness. Front. Oncol..

[52] Brożyna A.A., Jóźwicki W., Roszkowski K., Filipiak J., Slominski A.T. (2016). Melanin content in melanoma metastases affects the outcome of radiotherapy. Oncotarget.

[53] Slominski A., Zbytek B., Slominski R. (2009). Inhibitors of melanogenesis increase toxicity of cyclophosphamide and lymphocytes against melanoma cells. Int. J. Cancer.

[54] Ding H.Y., Chou T.H., Lin R.J., Chan L.P., Wang G.H., Liang C.H. (2011). Antioxidant and antimelanogenic behaviors of Paeonia suffruticosa. Plant Foods Hum. Nutr..

[55] Sarna M., Krzykawska-Serda M., Jakubowska M., Zadlo A., Urbanska K. (2019). Melanin presence inhibits melanoma cell spread in mice in a unique mechanical fashion. Sci. Rep..

[56] Wang X., Teng F., Kong L., Yu J. (2016). PD-L1 expression in human cancers and its association with clinical outcomes. Onco. Targets Ther..

[57] Lee J., Han Y., Wang W., Jo H., Kim H., Kim S., Yang K.M., Kim S.J., Dhanasekaran D.N., Song Y.S. (2021). Phytochemicals in Cancer Immune Checkpoint Inhibitor Therapy. Biomolecules.

[58] Tong Q., Wu Z. (2023). Curcumin inhibits colon cancer malignant progression and promotes T cell killing by regulating miR-206 expression. Clin. Anat..

[59] Cheon H., Holvey-Bates E.G., McGrail D.J., Stark G.R. (2021). PD-L1 sustains chronic, cancer cell-intrinsic responses to type I interferon, enhancing resistance to DNA damage. Proc. Natl. Acad. Sci. USA.

[60] Chen J., Chen R., Huang S., Zu B., Zhang S. (2021). Atezolizumab alleviates the immunosuppression induced by PD-L1-positive neutrophils and improves the survival of mice during sepsis. Mol. Med. Rep..

[61] von Delwig A., Altmann D.M., Charlton F.G., McKie N., Isaacs J.D., Holmdahl R., Robinson J.H. (2007). T cell responses to a non-glycosylated epitope predominate in type II collagen-immunised HLA-DRB1*0101 transgenic mice. Ann. Rheum. Dis..

[62] Chang M.R., Lee W.H., Choi J.W., Park S.O., Paik S.G., Kim Y.S. (2005). Antitumor immunity induced by tumor cells engineered to express a membrane-bound form of IL-2. Exp. Mol. Med..

[63] Jung D., Shin S., Kang S.M., Jung I., Ryu S., Noh S., Choi S.J., Jeong J., Lee B.Y., Kim K.S. (2022). Reprogramming of T cell-derived small extracellular vesicles using IL2 surface engineering induces potent anti-cancer effects through miRNA delivery. J. Extracell. Vesicles.

[64] Grabosch S., Bulatovic M., Zeng F., Ma T., Zhang L., Ross M., Brozick J., Fang Y., Tseng G., Kim E. (2019). Cisplatin-induced immune modulation in ovarian cancer mouse models with distinct inflammation profiles. Oncogene.

